# Bazhen Decoction Protects against Acetaminophen Induced Acute Liver Injury by Inhibiting Oxidative Stress, Inflammation and Apoptosis in Mice

**DOI:** 10.1371/journal.pone.0107405

**Published:** 2014-09-15

**Authors:** Erqun Song, Juanli Fu, Xiaomin Xia, Chuanyang Su, Yang Song

**Affiliations:** Key Laboratory of Luminescence and Real-Time Analytical Chemistry (Ministry of Education), College of Pharmaceutical Sciences, Southwest University, Chongqing, People's Republic of China; Yong Loo Lin School of Medicine, National University of Singapore, Singapore

## Abstract

Bazhen decoction is a widely used traditional Chinese medicinal decoction, but the scientific validation of its therapeutic potential is lacking. The objective of this study was to investigate corresponding anti-oxidative, anti-inflammatory and anti-apoptosis activities of Bazhen decoction, using acetaminophen-treated mice as a model system. A total of 48 mice were divided into four groups. Group I, negative control, treated with vehicle only. Group II, fed with 500 mg/kg/day Bazhen decoction for 10 continuous days. Group III, received a single dose of 900 mg/kg acetaminophen. Group IV, fed with 500 mg/kg/day Bazhen decoction for 10 continuous days and a single dose of 900 mg/kg acetaminophen 30 min before last Bazhen decoction administration. Bazhen decoction administration significantly decrease acetaminophen-induced serum ALT, AST, ALP, LDH, TNF-α, IL-1β, ROS, TBARS and protein carbonyl group levels, as well as GSH depletion and loss of MMP. Bazhen decoction restore SOD, CAT, GR and GPx activities and depress the expression of pro-inflammatory factors, such as iNOS, COX-2, TNF-α, NF-κB, IL-1β and IL-6, respectively. Moreover, Bazhen decoction down-regulate acetaminophen-induced Bax/Bcl-2 ratio, caspase 3, caspase 8 and caspase 9. These results suggest the anti-oxidative, anti-inflammatory and anti-apoptosis properties of Bazhen decoction towards acetaminophen-induced liver injury in mice.

## Introduction

Bazhen decoction, also recognized as eight-treasure decoction or eight precious decoction, was recorded in “A Repertory of Traumatology”. Bazhen decoction is a traditional Chinese medicine formula which related to a lot of symptoms, such as anemia, asthenia, breathlessness, chronic abscess, dizziness, fatigue, glare, irregular menstruation, lethargy, metrorrhagia, palpitations, poor appetite, anxiety, dull complexion, fatigue of the muscles, pale complexion, pale tongue, thin-white coating, fine-faint pulse and large-forceless-empty pulse. In traditional use, Bazhen decoction is a frequently-used prescription for the treatment of “qi and blood” deficiency. Bazhen decoction is a combination of Sijunzi decoction and Siwu decoction. The traditional use of Sijunzi decoction was to replenish or invigorate the intestine and stomach function, and promote the circulation of “qi and blood” [1]. Siwu decoction removes heat to cool blood, promotes blood flow and regulates menstruation, which has the best treatment effect for conditions of Yin blood deficiency [2]. In traditional Chinese medicine theory, “qi and blood” supplement each other, therefore, the combination of Sijunzi and Siwu decoction brings out the best in each other and appropriate for more severe conditions.

Acetaminophen (4-hydroxyacetanilide) is a clinical analgesic/antipyretic drug [3]. Although acetaminophen is safe at therapeutic doses, it causes hepatic injury in both human and experimental animals. In fact, acetaminophen overdose is a leading cause of drug-related acute liver failure [4]. The hepatotoxicity of acetaminophen has been related to many emergency hospital admissions, such as hepatitis, cirrhosis, and hepatic transplant and continues to be associated with high mortality [5].

The mechanism of acetaminophen-induced hepatotoxic effect is well known and has been extensively reviewed [6]–[9]. Cytochromes P450, mainly CYP2E1, catalyze acetaminophen to form corresponding reactive metabolite of acetaminophen, N-acetyl-p-benzoquinone imine, which is responsible for cytotoxicity, mutagenicity, and potential carcinogenicity. N-acetyl-*p*-benzoquinone imine easily reacts with cellular glutathione (GSH) and proteins. Besides protein modification, acetaminophen also contributes to the generation of reactive oxygen species (ROS) during its metabolism process. Antioxidant enzymes, such as superoxide dismutase (SOD), catalase (CAT), glutathione reductase (GR) and glutathione peroxidase (GPx), play critical roles in modulating the severity of acetaminophen-induced hepatotoxicity [10]. Lipid peroxidation (LPO) is also thought to linked with the initiation or progression of liver injury induced by acetaminophen [11]. Acetaminophen overdose trigger the transcriptional activation of pro-inflammatory factors, such as TNF-α, IL-1β and others in macrophages [12]. Previous studies have extensively demonstrated that antioxidants play important roles in scavenge ROS, inhibit oxidative stress and down-regulation of pro-inflammatory factors to against acetaminophen-induced toxicity [13]–[17].

The active components of Bazhen decoction were previously identified, which implied Bazhen decoction may possess anti-oxidative, anti-inflammatory and anti-apoptosis activities [18]. However, there is no direct evidence to support this hypothesis at this stage, which encourage us conduct the present work in order to investigate the protective effect of Bazhen decoction against acetaminophen-induced liver injury in mice. In addition, the possible mechanisms underlying these effects were also investigated.

## Materials and Methods

### Ethics statements

All animal experiments were performed according to the protocol approved by the Experimental Animal Ethical Committee of Southwest University. Animals were maintained in specific pathogen free laboratory under standard conditions of humidity (50±5%), temperature (25±2°C) in a 12 h light/12 h dark cycle. They were fed standard rodent chow and had free access to water, acclimatized for at least one week prior to use. All surgery was performed under ether anesthesia, and all efforts were made to minimize suffering.

### Materials and reagents

Acetaminophen (>98.0% HPLC) were purchased from Sigma-Aldrich Inc. Silymarin, 2,4-dinitrophenylhydrazine (DNPH) and 5,5′,6,6′-tetrachloro-1,1′,3,3′-tetraethylbenzimidazol-carbocyanine iodide (JC-1) dye were obtained from Aladdin Reagent Database Inc. Diagnostic kits used for the determination of ROS, TBARS, GSH, tumor necrosis factor-alpha (TNF-α), interleukin-1beta (IL-1β), SOD, CAT, GR and GPx, AST, ALT, ALP and LDH activities were obtained from the Nanjing Jiancheng Institute of Biotechnology (Nanjing, China). Rabbit polyclonal COX-2, iNOS, TNF-α, NF-κB, IL-1β, IL-6, Bcl-2, Bax, cytochrome c, caspase 3, caspase 8 and caspase 9 antibodies were purchased from Dingguo Biotechnology Co. Ltd (Beijing, China). All other chemicals used were of highest commercial grade.

### Preparation of Bazhen decoction

Herbs were purchased from an authentic herb supplier in the local market of Chongqing. Their Chinese names, English names, Latin names, family, part used, voucher numbers and daily adult dose (g) were presented in **Table 1**. The quality of these crude drugs was controlled and processed according to the Chinese Pharmacopoeia (2005). Radix Angelicae Sinensis (10 g), Rhizoma Chuanxiong (5 g), Radix Paeoniae Alba (8 g), Radix Rehmannia Libosch (15 g), Radix Ginseng (3 g), Rhizoma Atractylodis Macrocephalae (10 g), Poria cocos (8 g) and Radix Glycyrrhiza (5 g) were immersed in 500 mL distilled water and boiled for 30 min. The aqueous part was filtered. This procedure was repeated twice and the aqueous solutions were combined and lyophilized to yield brown pellet (5.7%, w/w). The dry residue was dispersed in distilled water before use.

**Table 1:** The compositions of Bazhen decoction.

Chinese names	English names	Latin names	Family	Part used	Voucher numbers	daily adult dose (g)
Danggui	Radix *Angelicae Sinensis*	Angelica sinensis (Oliv.) Diels	Umbelliferae	Root	20130205	10
Chuanxiong	Rhizoma *Chuanxiong*	Ligusticum striatum DC.	Apiaceae	Rhizome	20130306	5
Baishao	Radix *Paeoniae Alba*	Paeonia sterniana H.R.Fletcher	Paeoniaceae	Root	20130205	8
Shudi	Radix *Rehmannia Libosch*	Rehmannia glutinosa (Gaertn.) DC.	Plantaginaceae	Processed root tuber	20130306	15
Renshen	Radix *Ginseng*	Panax ginseng C.A.Mey	Araliaceae	Root	20130205	3
Baizhu	Rhizoma *Atractylodis Macrocephalae*	Atractylodes macrocephala Koidz	Compositae	Rhizome	20130205	10
Fuling	*Poria cocos*	Poria cocos wolf	Fungus	Sclerotium	20130401	8
Zhigancao	Radix *Glycyrrhiza*	Glycyrrhiza uralensis Fisch.	Leguminosae	Honeyed root and rhizome	20130205	5

### Animals and treatment

Male Kunming mice (20–24 g, 4 weeks old) were purchased from Chongqing Tengxin Biotechnology Co. Mice were randomly divided into five groups, each group consisting of 12 animals. (1) Negative control group, treated with vehicle only, (2) Bazhen group, 500 mg/kg/day Bazhen decoction was administered by intragastric gavage for 10 continuous days, (3) acetaminophen group, a single dose of 900 mg/kg acetaminophen (ip), (4) acetaminophen + Bazhen group, 500 mg/kg/day Bazhen decoction was administered by intragastric gavage for 10 continuous days and (5) acetaminophen + Silymarin group, 100 mg/kg/day Silymarin was administered by intragastric gavage for 10 continuous days. On the 10th day, a single dose of 900 mg/kg acetaminophen was injected (ip) 30 min before Bazhen decoction administration. After 12 h of acetaminophen injection, the animals were sacrificed under deep anesthesia. Blood was collected by cardiac puncture and allowed to clot for 45 min at room temperature. The livers were excised carefully, washed twice with saline, blotted dry on a filter paper, weighed and cut into two pieces. One halves were used for immunohistochemical analysis. Other halves were used for hepatic homogenate preparation.

### Cytosolic fraction isolation

Cell lysates were added to cytosolic extraction buffer, vortexed vigorously for 15 s. After incubation for 10 min on ice, cell lysates were centrifuged at 12,000 rpm for 10 min and the cytosolic fraction supernatants were transferred to new tubes and stored at −20°C until used.

### Biochemical analysis

The blood samples were centrifuged (600 g for 15 min) for serum collection. Serum analysis of various liver marker enzymes, such as ALT, AST, ALP and LDH activities were measured using assay kits according to the supplier specifications (Nanjing Jiancheng). Serum TNF-α and IL-1β were determined using commercially available enzyme linked-immunosorbent assay (ELISA) kits (Nanjing Jiancheng) according to the manufacturer's instructions.

Liver were rinsed in ice-cold physiological saline and homogenized in Tris-HCl buffer (0.01 M, pH = 7.4) to give a 10% homogenate. Homogenates were centrifuged at 3,000 rpm, 4°C for 10 min and supernatants were collected for further analysis. In the tissue samples, hepatic ROS, TBARS, GSH, SOD, CAT, GR and GPx levels were determined with commercial assay kits (Nanjing Jiancheng). Protein oxidative damage was evaluated by measuring protein carbonyl formation using 2,4-dinitrophenylhydrazine (DNPH) as a probe. Carbonyl content was determined by measuring the spectra of the supernatant at 365 nm (UV-2450, SHIMADZU, Japan). The results were expressed as nmol of DNPH incorporated/mg protein based on the molar extinction coefficient of 22,000 M^−1^ cm^−1^ for aliphatic hydrazones.

### Measurement of mitochondrial membrane potential (MMP)

Changes in mitochondrial membrane potential were assessed by staining with JC-1 dye. The homogenates were incubated with JC-1 staining buffer (5 µg/mL) for 20 min in the dark at 37°C. MMP were monitored by recording green (excitation/emission wavelength  = 485/510–525 nm) and red (excitation/emission wavelength  = 485/590 nm) fluorescence using a fluorescence microscope. Mitochondrial depolarization is indicated by an increase ratio in green fluorescence and decrease in red fluorescence.

### Immunohistochemical analysis of iNOS and COX-2

The paraffin-embedded sections were deparaffinized and rehydrated and were treated with a 1 mM EDTA buffer (pH = 9.0) in a microwave for 3 min for antigen retrieval. The following steps were followed the instruction of Histostain TM-Plus and diaminobenzidine substrate kits. Briefly, 3% H_2_O_2_ was used to block endogenous peroxidase activity. Nonspecific protein binding was blocked by normal goat serum. The rabbit polyclonal anti-COX-2 diluted 1∶50, and rabbit polyclonal anti-iNOS diluted 1∶50 were used as primary antibodies. Then, slide was incubated with biotin labeled goat-rabbit IgG and horseradish peroxidase-conjugated streptavidin for 1 h, respectively. Immunoreaction was visualized employing diaminobenzidine and counterstained by hematoxylin. Image was taken by a light microscopy (magnification, 200×, Nikon Eclipse Ti-SR).

### Western blotting

Proteins were separated by 10% SDS-PAGE electrophoresis and transferred onto nitrocellulose membrane. After blocking with 10% skimmed milk, membranes were incubated with primary polyclone rabbit antibodies TNF-α, NF-κB, IL-1β, IL-6, Bcl-2, Bax, cytochrome c, caspase 3, caspase 8 and caspase 9 at room temperature for 3 h. After washing three times with PBS, membranes were further incubated with secondary antibody conjugated with horseradish peroxidase (HRP) for 1.5 h. Finally, immune-reactive protein bands were detected by diaminobenzidine method. The relative density of protein expressions were quantitated by ImageJ software. Protein levels were standardized by comparison with β-actin.

### RNA isolation and real-time RT-PCR

Total cellular RNA was extracted from liver homogenate using innuPREP Micro RNA Kit according to the manufacturer's instructions (AJ Innuscreen GmbH). mRNA was reversely transcribed into cDNA (MMLV Reverse Transcriptase, Gibco/BRL) using oligo(dT) as primers and two-step real-time PCR was carried out using a LightCycler (Roche, Basel, Switzerland). Primers sequences were used as previous reported [19]. Bcl-2, 5′-GGGATGCCTTTGTGGAACTATA TG-3′ (forward), 5′-TGAGCAGCGTCTTCAGAGACA-3′ (reverse); Bax, 5′-GACACCTG AGCTGACCTTGGA-3′ (forward), 5′-GACACTCGCTCAGCTTCTTGGT-3′ (reverse); GAPDH, 5′-GCA CCG TCA AGG CTG AGA AC-3′ (forward), 5′-TGG TGA AGA CGC CAG TGGA-3′ (reverse). Thermocycler conditions included an initial holding period at 50°C for 2 min, then 94°C for 2 min, followed by 40 cycles of denaturation at 94°C for 30 s, annealing at 55°C for 15 s and extension at 72°C for 15 s. SYBR Green I fluorescence emission was monitored after each cycle, and mRNA levels were quantified using the second-derivative maximum method of the LightCycler software. The amount of each gene was expressed relative to the housekeeping gene, GAPDH. The products were separated by electrophoresis in 2% agarose gels and visualized by nucleic acid stain (Dingguo Biotechnology Co., Beijing, China) under ultraviolet light.

### Histopathological examination

Parts of the liver tissue were fixed by immersion in 4% paraformaldehyde and dehydrated. Sections of 5 µm thickness were taken and stained with hematoxylin and eosin (H&E). Slides were observed for histopathological changes using fluorescence microscope system (TE2000, Nikon, Japan) and representative images were presented.

### Statistical analysis

All experiments were repeated at least three times independently. All values were expressed as means ± SEM. The results were evaluated by one-way ANOVA and Tukey's multiple comparison tests. Differences were considered to be significant only for *p*<0.05.

## Results

### Effect of Bazhen decoction on serum biochemical parameters

Serum ALT/AST/ALP activities were assessed as biochemical markers of hepatic damage (**Table 2**). Bazhen decoction (500 mg/kg/day) treatment resulted in no change in serum ALT/AST/ALP activities compare to control group. A single dose of acetaminophen (900 mg/kg) showed hepatotoxic effect with the evidence of increased in ALT/AST/ALP serum activities. In contrast, Bazhen decoction treatment prevented acetaminophen-induced elevation of ALT/AST/ALP serum activities significantly. There has no significant difference between Bazhen and control group. Serum LDH activity is a marker of generalized tissue damage as well. Our result showed a significant increase in acetaminophen group (4356.1±134.5 U/L) when compared to control group (877.4±45.6 U/L), however, acetaminophen + Bazhen group showed reduced LDH activity (1032.5±99.5 U/L, *p*<0.001 *vs* acetaminophen group). Silymarin also showed protective effect may imply similar protective mechanism.

**Table 2:** Effects of Bazhen decoction on serum biochemical parameters in mice intoxicated with acetaminophen.

group	ALT (U/L)	AST (U/L)	ALP (U/L)	LDH (U/L)
control	43.2±7.0	50.3±5.9	120.1±11.9	877.4±45.6
Bazhen	35.8±6.9	41.1±8.2	123.4±22.1	822.4±62.0
acetaminophen	767.1±152.0***	1003.6±181.4***	480.3±35.9***	4356.1±134.5***
acetaminophen + Bazhen	122.2±38.4^###^	279.0±60.2^###^	184.8±24.7^###^	1032.5±99.5^###^
acetaminophen + Silymarin	81.8±15.6^###^	110.5±22.6^###^	167.8±22.9^###^	908.5±113.7^###^

Values are expressed as means ± SD of 12 animals in each group. Significance difference were ***p<0.001 as compared with the control; ^###^ *p*<0.001 as compared with acetaminophen group.

### Effect of Bazhen decoction on serum cytokine expressions

**Figure 1:**
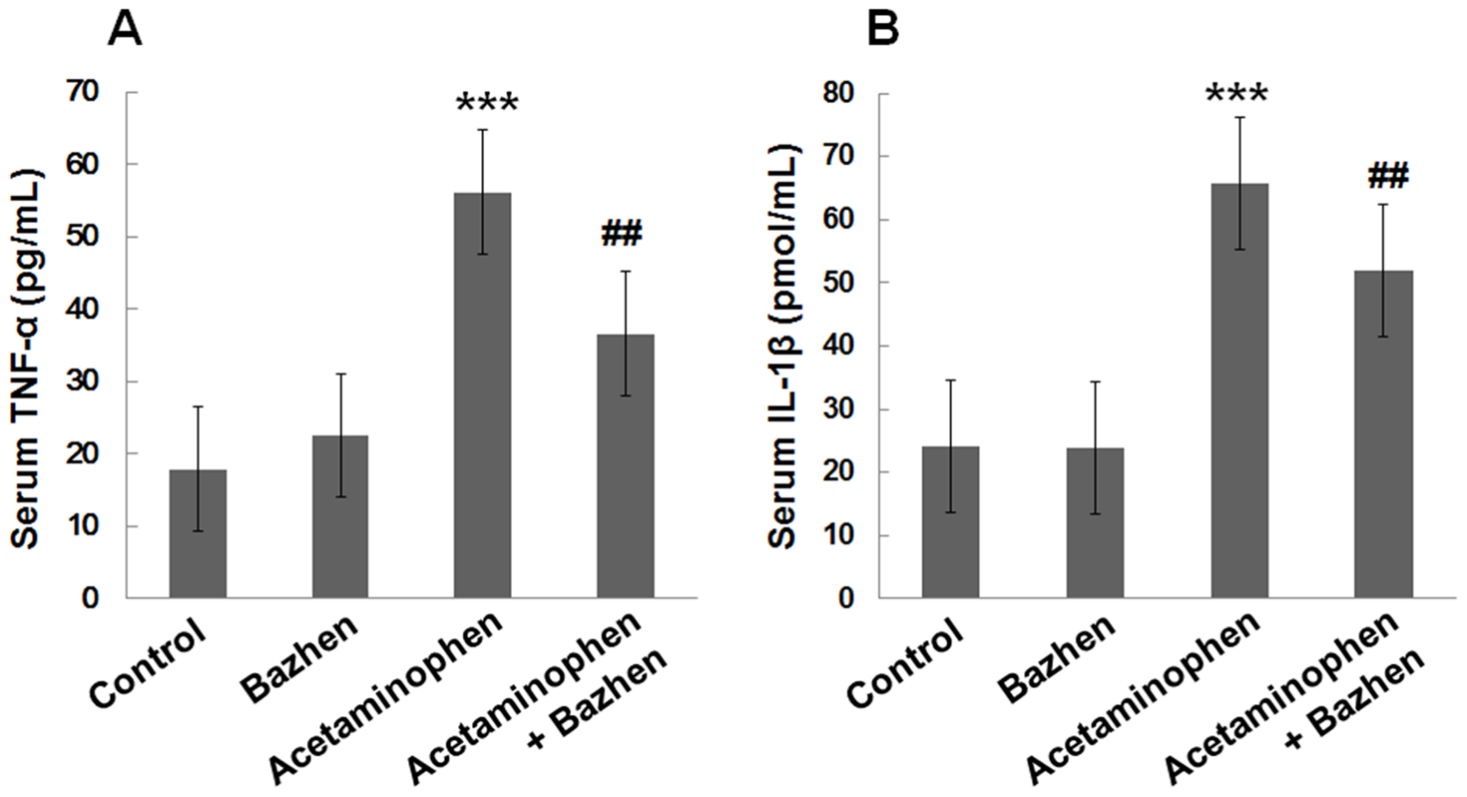
Effect of Bazhen decoction on serum TNF-α and IL-1β levels in acetaminophen intoxicated mice. (**A**) TNF-α and (**B**) IL-1β. Values are expressed as means ± SD of 12 animals in each group. Significance difference were ***p<0.001 as compared with the control; ^##^ *p*<0.01 as compared with acetaminophen group.

To investigate the effect of Bazhen decoction on the modulation of inflammatory cytokines, serum TNF-α and IL-1β were determined. As shown in **Figure 1**, Acetaminophen-administration significantly increased levels of TNF-α (56.1±11.2 pg/mL) and IL-1β (65.7±4.2 pmol/mL) compared with their levels in normal mice (17.9±2.4 pg/mL and 24.1±4.9 pmol/mL, respectively), suggesting that severe inflammatory reaction had been conducted. However, Bazhen decoction significantly inhibited serum TNF-α and IL-1β levels (36.6±4.7 pg/mL and 52.0±4.9 pmol/mL, respectively, *p*<0.001), compared with acetaminophen group.

### Effect of Bazhen decoction on hepatic non-protein oxidative stress markers

**Figure 2:**
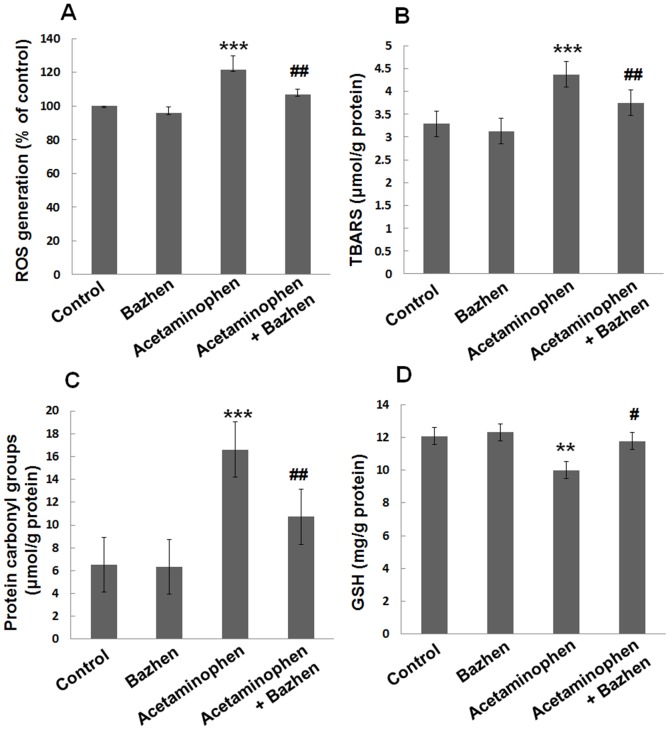
Effect of Bazhen decoction on hepatic ROS, TBARS, protein carbonyl group and GSH levels in acetaminophen intoxicated mice. (**A**) ROS, (**B**) TBARS, (**C**) protein carbonyl group and (**D**) GSH. Values are expressed as means ± SD of 12 animals in each group. Significance difference were ***p<0.001 or **p<0.01 as compared with the control; ^##^ *p*<0.01 or ^#^ *p*<0.05 as compared with acetaminophen group.

Acetaminophen administration showed increased levels of ROS (121.3±8.5% vs. 100%, *p*<0.001), TBARS (4.4±0.1 vs. 3.3±0.3 µmol/g protein, *p*<0.001) and protein carbonyl group (16.6±2.1 vs. 6.5±1.2 µmol/g protein, *p*<0.001) in the mice liver, as compared to control group, **Figure 2A, 2B and 2C**. However, acetaminophen + Bazhen group had significantly inhibition of ROS (106.6±3.4%), TBARS (3.7±0.2 µmol/g protein) and protein carbonyl group (10.7±3.0 µmol/g protein), *p*<0.001 respectively.

The endogenous antioxidant, GSH level in hepatic tissue was decreased significantly (*p*<0.01) in acetaminophen group (10.0±0.70 mg/g) as compared to control group (12.1±0.31 mg/g), while Bazhen decoction treatment significantly (*p*<0.05) reversed this acetaminophen-induced GSH reduction (11.8±0.55 mg/g), **Figure 2D**.

### Effect of Bazhen decoction on hepatic antioxidant enzyme activities

**Figure 3:**
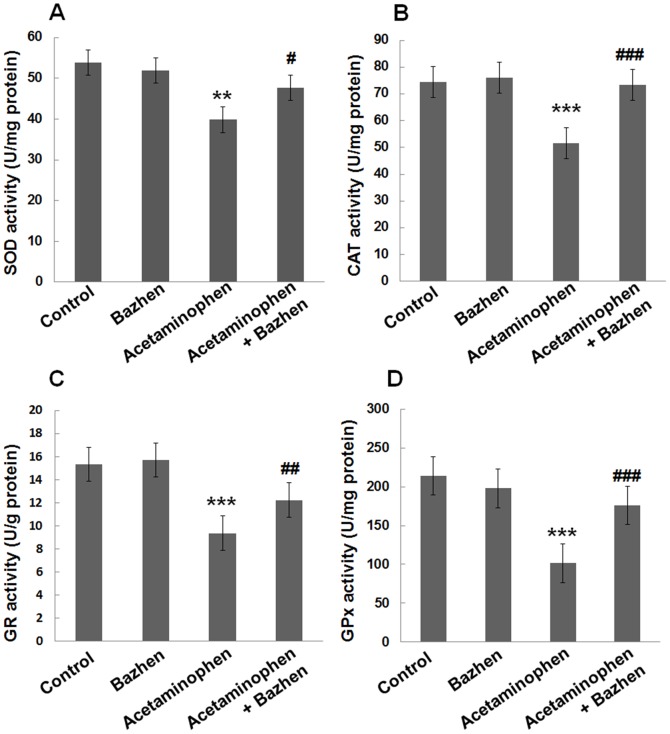
Effects of Bazhen decoction on the hepatic antioxidant enzymes activities in mice intoxicated with acetaminophen. (**A**) SOD, (**B**) CAT, (**C**) GR and (**D**) GPx. Values are expressed as means ± SD of 12 animals in each group. Significance difference were ***p<0.001 or **p<0.01 as compared with the control; ^###^ *p*<0.001, ^##^ *p*<0.01 or ^#^ *p*<0.05 as compared with acetaminophen group.

Hepatic SOD (39.8±4.1 vs. 53.8±4.0 U/mg protein), CAT (51.6±4.5 vs. 74.5±6.1 U/mg protein), GR (9.4±0.8 vs. 15.3±1.0 U/g protein) and GPx (101.6±10.7 vs. 213.8±15.5 U/mg protein) activities were significantly reduced in acetaminophen-intoxicated mice, compared to normal group (*p*<0.001), as shown in **Figure 3**. However, pretreatment with Bazhen decoction significantly enhanced SOD (47.6±3.0 U/mg protein), CAT (73.3±4.4 U/mg protein), GR (12.2±1.0 U/g protein) and GPx (175.9±11.0 U/mg protein) activities compared with those of acetaminophen-intoxicated mice. Administration of Bazhen decoction alone has no significant effect on enzymatic antioxidants.

### Effect of Bazhen decoction on inflammatory response in mouse livers

**Figure 4:**
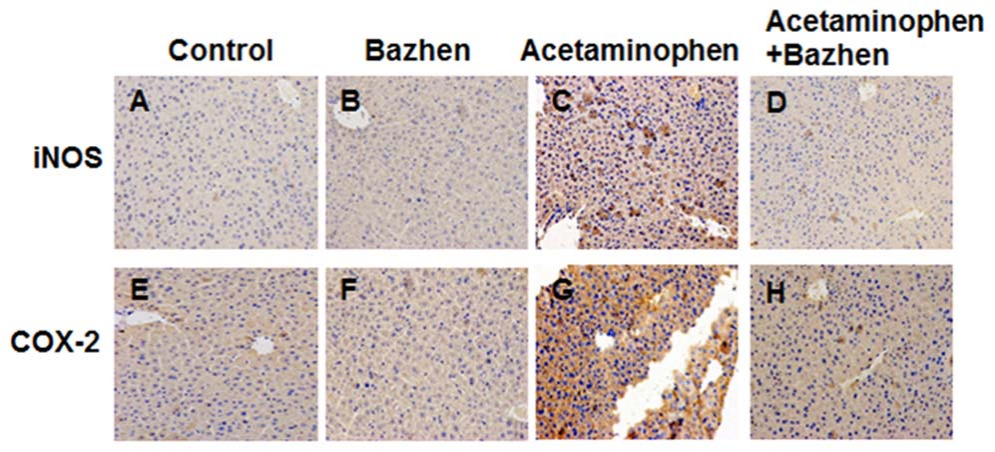
Effects of Bazhen decoction on acetaminophen-induced immunohistochemical stain of iNOS and COX-2 expression in liver tissue. Typical images were shown for iNOS expression in (**A**) control, (**B**) Bazhen, (**C**) acetaminophen and (**D**) acetaminophen + Bazhen group, and COX-2 expression in (**E**) control, (**F**) Bazhen, (**G**) acetaminophen and (**H**) acetaminophen + Bazhen group. Original magnification 200×.

We observed the effect of Bazhen decoction on hepatic iNOS and COX-2 levels using immunohistochemical assay. The livers of control mice did not show substantial iNOS and COX-2 immunopositivity (**Figure 4A and 4E**), and the animals receiving Bazhen decoction alone were similar to control (**Figure 4B and 4F**). However, acetaminophen-treatment dramatically increased iNOS and COX-2 immunopositivity in the livers compared with control (**Figure 4C and 4G**), which was attenuated by Bazhen decoction administration (**Figure 4D and 4H**).

**Figure 5:**
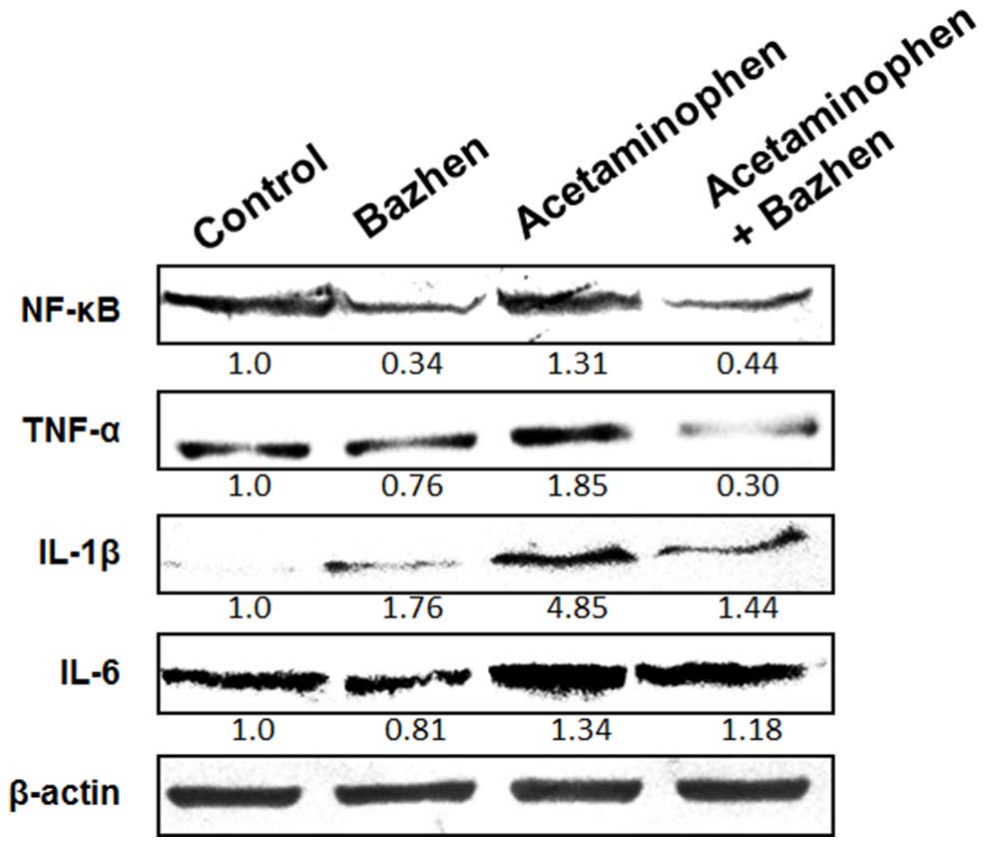
Effects of Bazhen decoction on the hepatic NF-κB, TNF-α, IL-6 and IL-1β expressions in mice intoxicated with acetaminophen. Data were normalized to β-actin used as control.

Western blotting results indicated the protective effect of Bazhen decoction on the expression of TNF-α, NF-κB, IL-1β and IL-6 in hepatic tissues. All these proteins associated with inflammation showed significantly up-regulated in acetaminophen group, but down-regulated with Bazhen decoction administration (**Figure 5**).

### Effect of Bazhen decoction on apoptosis features in mouse livers

**Figure 6:**
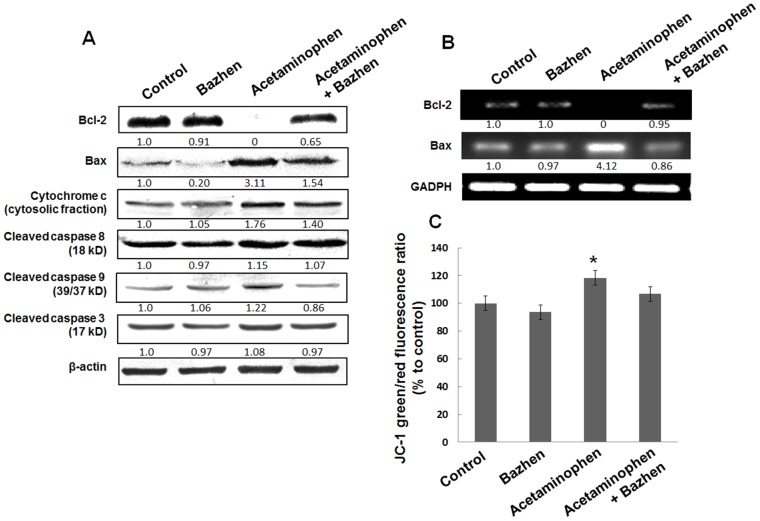
Effects of Bazhen decoction on apoptotic features in mice intoxicated with acetaminophen. (**A**) Western blotting of Bcl-2, Bax, cytochrome c (cytosolic fraction), cleaved caspase 8, 9 and 3 expressions. Data were normalized to β-actin used as control, (**B**) mRNA expression of Bcl-2 and Bax. GAPDH was used as internal control and (**C**) Fluorimetric quantified the green to red fluorescence ratio of JC-1. The value of control group was set as 100%.

The decreased anti-apoptotic gene Bcl-2 and increased pro-apoptotic gene Bax were showed in acetaminophen-intoxicated mice liver, however, Bazhen decoction reverted these changes significantly (**Figure 6A**). It is interesting to notice that acetaminophen showed significant effect on mRNA levels of Bcl-2 and Bax, which is coordinate with their protein expressions (**Figure 6B**). To gain a better understanding of the underlying mechanism, we determined the effect of Bazhen decoction on acetaminophen-induced loss of MMP. As shown in **Figure 6C**, the loss of MMP was observed after treatment with acetaminophen. However, Bazhen decoction reversed this effect, although no statistical significance was found between (Bazhen and acetaminophen + Bazhen group). In addition, the expressions of cleaved (activated) caspase family protein following the administration of acetaminophen were higher than in the control group. On the contrary, Bazhen administration attenuated the enhanced expression of activated caspase family proteins.

**Figure 7:**
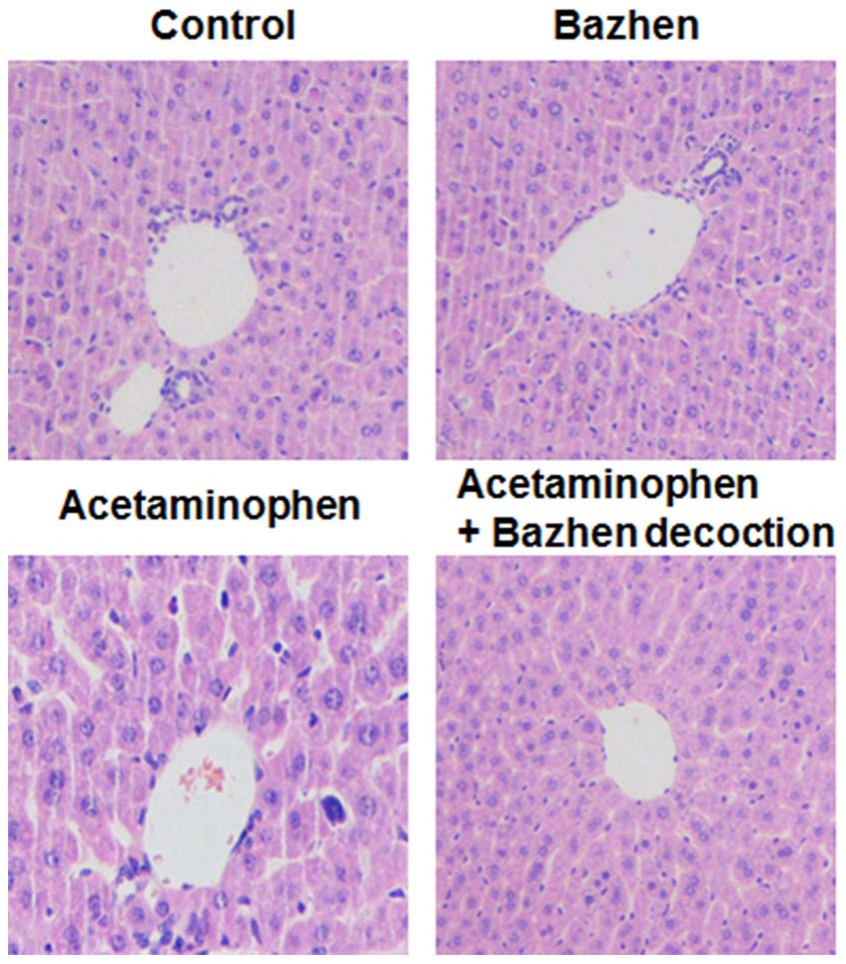
Effect of Bazhen decoction on acetaminophen-induced histopathological change in the liver tissue. Original magnifications were 200x.

Histopathological changes were presented in **Figure 7** to support the effect of Bazhen decoction on acetaminophen-induced liver injury. Liver section in acetaminophen group showed cytoplasmic vacuolation and degeneration of hepatocytes, along with necrosis and inflammatory cells, compare with control group. Bazhen effectively limits the acetaminophen-induced acute liver injury.

## Discussion

Early study identified the metabolites of Bazhen decoction from rat feces using a high performance liquid chromatography/mass spectrometry (HPLC/MS) technique [18]. A total of 29 major fragments were found and 19 chemical constituents were identified in Bazhen decoction, including Paeoniflorin, Atractylenolide II, Benzoyl-paeoniflorin, Liquiritin, Pachymic acid, Ginsenoside Re, Ginsenoside Rg_1_, Ginsenoside Rb_1_, Ginsenoside Rd, Glycyrrhizic acid, Paeonimetabolin I, Aucubinine A, 20-O-β-D-glucopyranosyl-20 (S)-protopanaxadiol, 20 (S)-Protopanaxatriol, 16 α-hydroxy acids perforation, 20 (S)-Protopanaxadiol, Licorice saponine, 18 β-Glycyrrhetinic acid and Ginsenoside Rh_1_. Liu *et al* identified three types of active components, ginsenoside, flavonoid and triterpenoid in Sijunzi decoction, using HPLC-coupled tandem mass spectrometry (HPLC/MS^n^) [1]. Su *et al* identified eighty-four components in Siwu decoction, including ten organic acids, thirty glycosides, fourteen lactones, eighteen flavonoids and eleven alkaloids using ultra-high performance liquid chromatography coupled time-of-flight mass spectrometry (UPLC-QTOF-MS^n^) and HPLC-DAD [20]. Many investigators illustrated these constituents have promising anti-oxidative and anti-inflammatory activities. For example, Paeoniflorin protects human EA.hy926 endothelial cells against gamma-radiation induced oxidative injury by activating the NF-E2-related factor 2/heme oxygenase-1 pathway and it also protect against LPS-induced liver inflammation [21], [22], liquiritin exerts neuroprotective effect against focal cerebral ischemia/reperfusion in mice *via* its anti-oxidative properties [23], pachymic acid improves bone disturbance against AH plus-induced inflammation in MC-3T3 E1 cells for its anti-oxidative property [24], ginsenoside analogs have great potential on anti-oxidative and anti-inflammatory applications for Alzheimer's disease and other neurodegenerative disorders [25]–[27]. Paeoniflorin showed effective effect on the protection of hypoxia-induced apoptosis in endothelial cells [28]. These results strongly suggested the protective effect of Bazhen decoction.

Liver is a major organ for metabolism, which has a number of functions including glycogen storage, decomposition of red blood cells, plasma protein synthesis and detoxification. However, it is also an important target for the toxicity of drugs in terms of oxidative stress. The accumulation of ROS, including superoxide, hydroxyl, and hydrogen peroxide cause oxidative stress and DNA, proteins and lipids damages. Acetaminophen-induced hepatotoxicity is characterized as increased oxidative stress and the massive impairment of antioxidant defense systems [7]. Lipid peroxidation is also associated with acetaminophen-induced toxicity [29]. The level of TBARS and protein carbonyl group, which indicate the degree of lipid and protein peroxidation, respectively. On the other hand, cellular antioxidant enzymes, including SOD, CAT, GR and GPx were impaired with acetaminophen treatment [30]. The present study showed TBARS/protein carbonyl group level was significantly increased and the activities of SOD/CAT/GR/GPx in mice liver were dramatically decreased by the treatment of acetaminophen. Interestingly, Bazhen decoction could markedly inhibit TBARS and protein carbonyl group formations and restore the activities of those antioxidant enzymes in the livers of acetaminophen-treated mice. In addition, Bazhen decoction remarkably reduced serum ALT/AST/ALP/LDH levels, which coincided with the reduction of acetaminophen-induced oxidative injury. These results, for the first time, indicate that Bazhen decoction protected the liver against oxidative stress through the inhibitions of ROS production and lipid/protein peroxidation.

Inflammation has been considered as a protective reaction to against invading pathogens or chemicals in order to maintain body health [31]. However, the inflammatory process, also mediate the progression of tissue damage and resulting in a number of neurodegenerative diseases, *e.g.*, Alzheimer's disease and Parkinson's disease [32]. Inhibition of inflammatory cytokine production play a central role in therapeutic potential for treatment of inflammatory diseases. During inflammation, many cytokines were up-regulated and accumulated in the liver, among them, TNF-α [33] and IL-1β [34] have been recently implicated as critical mediators of acetaminophen-induced hepatotoxicity. In the current study, we demonstrated that Bazhen decoction exhibits anti-inflammatory properties by inhibiting the activities of TNF-α and IL-1β in serum and the expression of iNOS, COX-2, TNF-α, NF-κB, IL-1β and IL-6 in liver. Inflammation mediated damage also attributed to over-production iNOS and COX-2 [35]. iNOS is essential in immune and inflammatory response to a variety of xenobiotics, which reflect the degree of inflammation. COX-2, an inducible isoform of cyclooxygenases, has been associated with inflammatory response in many different diseases [36]. Both iNOS and COX-2 were up-regulated in response to inflammation challenge, have been shown to be the major factors in mediating inflammatory processes. These results implied that the protective effect of Bazhen decoction may also due to its anti-inflammatory ability.

Massive evidences demonstrated acetaminophen induced translocation of Bcl-2 family proteins [37], release of cytochrome c [38] and positive cells staining for TUNEL assay [39]. Therefore, we also investigated the effect of Bazhen decoction on acetaminophen-induced apoptotic signals. Our results revealed that acetaminophen down-regulated Bcl-2 and up-regulated Bax in hepatocytes, which is in line with previous studies [40], [41]. Accordingly, acetaminophen-induced release of cytochrome c from the mitochondria to the cytoplasm with the breakdown of MMP, and the activation of caspase 8, caspase 9 and caspase 3 were showed in previous works [41]–[43]. However, we first showed that Bazhen decoction administration dramatically reversed acetaminophen-induced elevation of Bax/Bcl-2 ratio on protein and mRNA level respectively. It also alleviated the release of cytochrome c, as well as the activation of caspase 8, caspase 9 and caspase 3. It is worth to mention that only slight up-regulation of cleaved caspase family proteins were detected in acetaminophen-treated animals. Some arguments claimed that acetaminophen-induced Bax translocation or cytochrome c release are not specific for apoptosis features, and there has no evidence for caspase activation [37], [38], therefore, acetaminophen-induced liver injury may *via* necrosis rather than apoptosis mechanism. Indeed, histopathological examination showed necrosis and inflammatory cells in the current study. However, controversial results also presented, acetaminophen was found not only down-regulated Bcl-2 and up-regulated Bax, release of cytochrome c, but also the activation of caspase 9/3 [40], [41]. Interestingly, Ray et al first reported that 40% of hepatocytes actually die by apoptosis after acetaminophen overdose [44], however, a detailed morphological study clearly demonstrated that >95% of injured hepatocytes die through oncotic necrosis in vivo [39]. Therefore, the mechanism of acetaminophen induced cell death may base on their corresponding experimental model, and need further investigation.

In conclusion, our results confirmed the anti-oxidative, anti-inflammatory and anti-apoptosis effects of Bazhen decoction in acetaminophen-treated mice. The protective mechanism of Bazhen decoction is, at least in part, associated with the up-regulation of anti-oxidative enzyme activities and down-regulation of pro-inflammatory factors resulting from acetaminophen administration. In addition, Bazhen decoction disrupted the mitochondrial apoptotic pathway by up-regulating Bcl-2 and down-regulating Bax expression, preventing caspase 8, caspase 9 and caspase 3 activation. Taking together, Bazhen decoction might be used as a potential therapeutic strategy to prevent acute liver injury encountered with acetaminophen overdose. Furthermore, our results indicated the possible usage of Bazhen decoction in the treatment of toxic acute liver failure.
